# Sleep medicine and chronobiology education among Brazilian medical students

**DOI:** 10.1590/1414-431X2024e14147

**Published:** 2024-12-13

**Authors:** Y.C. Novais, J. Borges, F.A. Ferreira-Moraes, E.K. Tamura

**Affiliations:** 1 Grupo de Pesquisa em Cronobiologia, Departamento de Ciências da Saúde, Universidade Estadual de Santa Cruz, Ilhéus, BA, Brasil

**Keywords:** Sleep education, Biological rhythms, Brazilian medical students, Medical curriculum, Sleep health

## Abstract

Chronobiology and sleep medicine are pivotal disciplines for understanding human health. Additionally, the lack of training in medical schools exacerbates the underdiagnosis and undertreatment of sleep disorders. This study investigated the exposure of Brazilian medical students to chronobiology and sleep medicine during their undergraduate education and assessed their knowledge in these areas. The study was conducted between December 2021 and June 2022 through the administration of an online questionnaire (Google Forms) to medical students in the final two years of undergraduate studies. The questionnaire gathered student data (i.e., sex, age, and educational institution), research data (stage in the medical program with exposure to chronobiology or sleep medicine), and responses to a questionnaire on basic knowledge of sleep medicine and chronobiology (adapted from Assessing Sleep Knowledge in Medical Education - ASKME). A total of 240 students from medical schools in Brazil participated, 4.6% of whom reported no exposure to either subject, with exposure declining as students progressed through the curriculum. Only 3.7% reported specific disciplines covering these topics. Over half of the students encountered learning barriers, such as limited curriculum time. Despite these challenges, the accuracy rate in responses regarding chronobiology and sleep medicine was 79.75%, positively correlating with exposure level and knowledge retention. This study underscores the urgent need for enhanced education in sleep medicine and chronobiology within Brazilian medical schools. It offers insights into the current landscape of sleep medicine education in Brazil and lays groundwork for future efforts to integrate these essential subjects into medical school curricula.

## Introduction

Sleep medicine and chronobiology are two prominent fields of knowledge that encompass vital aspects of human health, namely sleep and other biological rhythms. The recent recognition of these interconnected sciences was underscored by the prestigious 2017 Nobel Prize in Physiology or Medicine, bestowed upon Jeffrey C. Hall, Michael Rosbash, and Michael W. Young for their groundbreaking contributions to understanding the circadian rhythm (1).

Chronobiology is the science of the intricate biological rhythms governing various organisms. Its primary focus is the study of the impact of time on biological events and the internal biological clocks that regulate these rhythms. Over the past several decades, chronobiology has evolved into a multidisciplinary field of great interest, particularly in the realm of general medicine (2). Besides the sleep-wake cycle, other biological rhythms include heart rate, respiratory rate, menstrual cycle, and pulsatile hormonal secretion (3).

Among the circadian rhythms (around 24 h), the sleep-wake rhythm emerges as the most extensively studied and discussed in relation to human health, with a substantial portion of the global population experiencing sleep-related issues (2,4). Given that a multitude of human functions, both physical and cognitive, display circadian rhythmicity, it intuitively follows that disturbances in the endogenous machinery regulating these oscillations could lead to physical and mental symptoms, as well as pathological conditions (5).

Throughout history, the medical literature had a limited focus on sleep disorders, primarily encompassing disturbances perceived as troublesome by those affected, such as insomnia (6). Other sleep disorders arising from physiological system malfunctions during sleep, such as sleep-related respiratory disorders, remained largely unknown or overlooked until the advent of sleep monitoring techniques, particularly in the second half of the 20th century (7). The establishment of sleep medicine as an independent medical specialty, along with its diagnostic procedures and therapeutic strategies, became possible thanks to seminal discoveries in neurophysiology and basic sleep research (8). These milestones marked a crucial turning point in the field of sleep medicine, enabling it to evolve into a specialized discipline with its own unique contributions to the medical landscape.

Over the past three decades, several studies have highlighted a significant gap in education on sleep and chronobiology in medical curricula worldwide. For example, Romiszewski et al. (9) demonstrated that education on sleep remains insufficient in medical schools in the United Kingdom, even after twenty years of recognizing the importance of the subject. Similar studies conducted in the United States (10,11), Saudi Arabia (12), China (13), and Lebanon (14) support these observations and highlight the urgent need to integrate these topics into medical curricula.

Previous data from our research group have indicated a limited interaction between chronobiology and psychology in the country (15). Additionally, despite the fact that most psychologists report an increase in patients with sleep-related issues, a lack of familiarity with basic concepts of chronobiology and sleep science has been identified among psychologists, likely because 75.97% of them had no academic contact with biological rhythms during their training (15). This suggests that education needs to be expanded not only in medicine but also in other health fields. Similarly, chronobiology is not typically included in the education of biology or medical students in the majority of European countries (16).

The current situation regarding the inclusion of these subjects in the curriculum of Brazilian medical schools remains unclear. However, we hypothesize that there is a significant underrepresentation of these topics, which may contribute to the underdiagnosis and undertreatment of sleep disorders in the country. Furthermore, this study can serve as a foundation for future research aimed at advancing the study of sleep medicine and chronobiology within medical schools, due to its contribution in providing an overview of the Brazilian landscape concerning the incorporation of these subjects into the curriculum. Thus, our objective was to assess the exposure of Brazilian medical students in their final two years of undergraduate medical education to the fields of chronobiology and sleep medicine and to evaluate their general knowledge in these areas.

## Material and Methods

### Study design and period

A cross-sectional study was conducted from December 1, 2021 to June 30, 2022. This study used self-reported online questionnaires and was conducted in accordance with the provisions of the Declaration of Helsinki and approved by the Ethical Committee of the State University of Santa Cruz (CEP-UESC) under Certificate of Presentation for Ethical Appreciation (CAAE; #52462921.0.0000.5526). Written informed consent was obtained from all the participants. The Google Forms platform was used to obtain informed consent and responses.

### Sampling and recruitment

The research targeted students in the final two years of undergraduate medical programs at Brazilian medical schools that are duly registered and listed on the e-MEC portal maintained by the Ministry of Education. The sample was obtained by contacting undergraduate medical students in social media networks associated with medical schools, including athletic clubs, academy centers, extension projects, and similar platforms. In this initial communication, the research title, the desired participant profile, and the researchers contact information were provided. The aim was to emphasize the significance of their participation and address any inquiries.

The inclusion criteria were outlined as follows: 1) being an undergraduate medical student at a Brazilian educational institution; 2) enrollment in the last two years of the program; and 3) specifying whether they were students in the fifth or sixth year.

Upon their agreement to participate in the study, data collection was conducted using a self-administered questionnaire within a virtual environment. The questionnaire was hosted on a freely accessible platform (Google Forms), and participants were asked to share the link with their classmates. Access was granted after participants provided an email for identification purposes and agreed to the free and informed consent form. Once these steps were completed, participants were granted access to the subsequent stage, which encompassed the questionnaire.

According to the 2020 Medical Demographics study in Brazil, the number of undergraduate medical students participating in the National Student Performance Exam (ENADE) in higher education institutions was 20,618 in 2019 (17). This number was used to calculate the sample size, assuming a confidence level of 95% and a margin of error of 5%, which resulted in a minimum of 240 students. All study participants volunteered to participate in the study, resulting in the recruitment of 243 students. One duplicate response, one individual outside the target audience, and one individual who did not provide responses to any items on the form were excluded from the analysis. Hence, a total of 240 samples were included and analyzed for the study.

### Questionnaire

The online questionnaire was developed by the authors in Portuguese (English version: Supplementary Table S1; original Portuguese version: Supplementary Table S2) and was structured into three sections.

I. Student data: age (18-24 or >24), sex, academic year (5th or 6th year), the Brazilian state where the medical school is located, and the name of the institution.

II. Research data: exposure to chronobiology and sleep medicine across basic, clinical, and internship cycles. This section was created by the researchers to identify the subjects’ knowledge of sleep medicine and chronobiology within their overall medical school curriculum, rather than within specific course.

III. Questionnaire on Basic Knowledge in Sleep Medicine and Chronobiology: This section included ten questions adapted from the Assessing Sleep Knowledge in Medical Education - ASKME (18). The ASKME questions selected were intended to be the most general questions regarding the sleep-wake cycle. The questionnaire was designed to evaluate long-term memory rather than highly specific technical knowledge or recent memory. It examined the student's exposure to the topic during their undergraduate studies using true, false, or “don't know” questions (18).

### Statistical analysis

The questionnaires were checked for completeness, coded, and entered into a Microsoft Excel table and then exported to SPSS (v.22, IBM, USA) for analysis. Categorical variables are reported as frequencies and percentages. In the analysis related to the internship cycle, we exclusively considered students in their final year (6th year). Bivariate analysis was used primarily to check the association of independent variables with the dependent variable (≥80% of correct answers in the questionnaire). Multiple logistic regression models were conducted, using age, sex, country region, and academic year as covariates for all other independent variables with the aim of analyzing potential confounding effects. The first category of each independent variable was considered as the reference group. The variables with a significant association were identified based on odds ratio (OR) with P-value ≤0.05. Additionally, Spearman's correlation was used to assess the relationship between the number of cycles in the medical program during which students were exposed to chronobiology or sleep medicine and the percentage of correct answers regarding basic chronobiology and sleep medicine knowledge.

## Results

A total of 240 students responded to the questionnaires. The most represented age group was 18 to 24 years, corresponding to 51.8% of the total. Additionally, 63.2% of the participants were women (n=152). Responses were obtained from students across the country, representing 96 institutions, with higher representation from the northeast (36.5%) and southeast (19.9%) regions. There was a maximum of 11 responses from two universities, namely the Universidade Estadual de Santa Cruz (UESC) and the Universidade Federal do Amazonas (UFAM), while the other universities had a smaller and similar number of responses. Regarding their stage in medical school, 153 students were in the 5th year and 87 students were in the 6th and final year (Table 1).

**Table 1:** Descriptive characteristics of the study population.

Variable	Frequency	Percentage (%)
Age (years)		
18-24	126	52.5
>24	114	47.5
Gender		
Women	152	63.3
Men	88	36.7
Country region		
North	45	18.7
Northeast	87	36.3
Midwest	25	10.4
Southeast	48	20.0
South	35	14.6
Academic year		
5th year	153	63.8
6th year	87	36.2

Sequentially, we examined whether students were exposed to sleep medicine or chronobiology throughout their medical undergraduate disciplines (Table 2). Our observations revealed a strong exposure during the basic cycle (first two years of the undergraduate medical program in Brazil; 87.5%), with a gradual decline during the clinical cycle (third and fourth years of the undergraduate medical program in Brazil; 77.1%) and the internship phase (last two years of the undergraduate medical program in Brazil; 65.5%). Additionally, 11 respondents (4.6% of the total) reported having had no contact with the subject throughout their entire undergraduate education, while 229 (95.4% of the total) had some exposure at some point. Notably, 62.1% had coursework related to these issues during all cycles of the medical program (Table 2).

**Table 2:** Contact with chronobiology and sleep medicine during the different periods of medical training.

Variable	Studies in chronobiology and sleep medicine	
	No	Yes
Basic cycle (n=240)	30 (12.5%)	210 (87.5%)
Clinical cycle (n=240)	55 (22.9%)	185 (77.1%)
Internship (n=87)	30 (34.5%)	57 (65.5%)
Any cycle (n=240)	11 (4.6%)	229 (95.4%)
All cycles (n=87)	33 (37.9%)	54 (62.1%)

**Figure 1:**
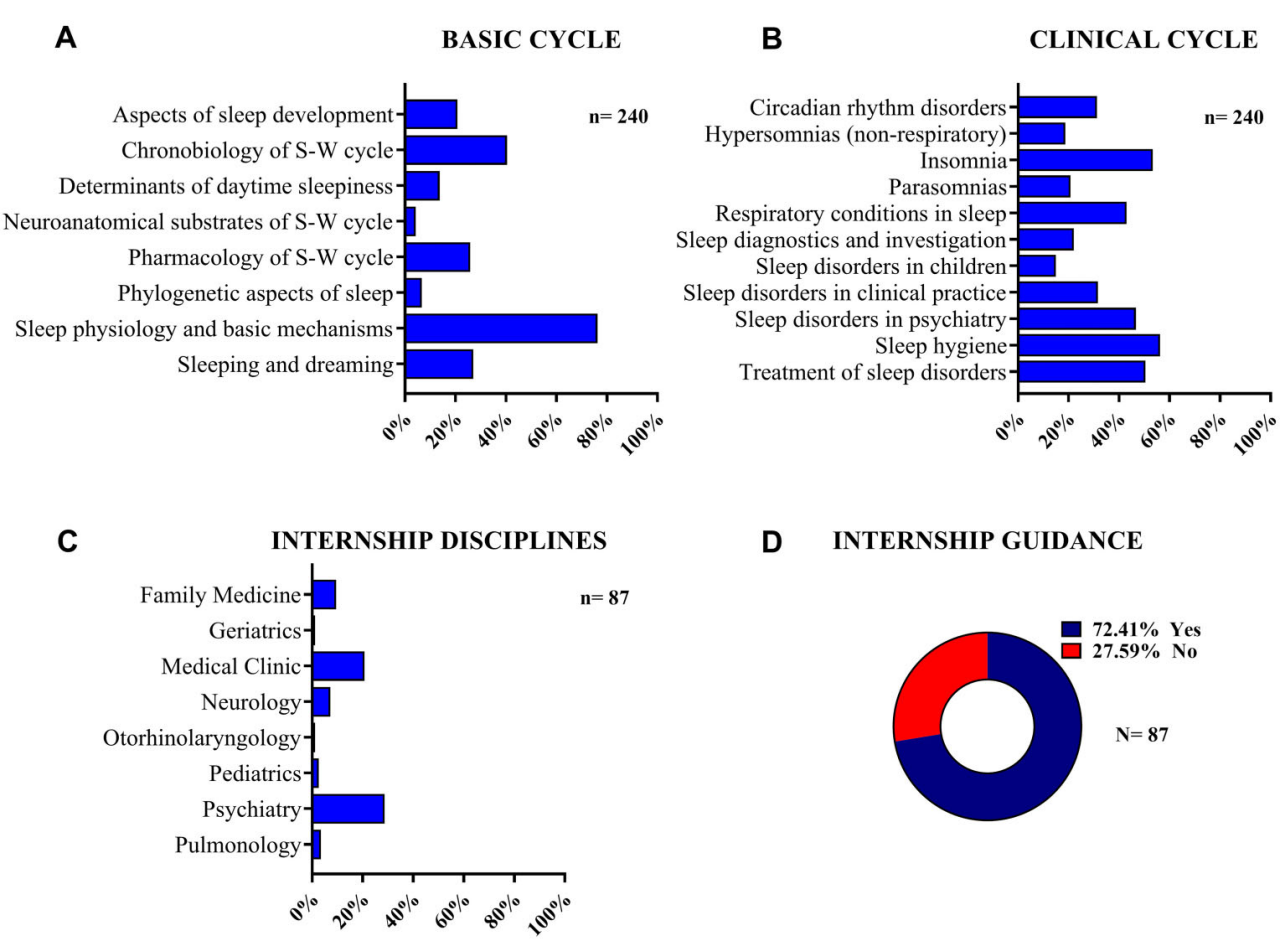
Exposure to chronobiology and sleep medicine in the stages of the medical program. The images show the frequency (%) of students who had academic contact with each topic and subtopic related to chronobiology and sleep medicine, studied during the basic (**A**), clinical (**B**), and internship cycles (**C**). **D**, Frequency of students (%) who received guidance during the internship phase to conduct patient history taking (anamnesis) and diagnosis regarding sleep-related aspects. The number of students is indicated in each graph. S-W: sleep-wake.

The students also selected the disciplines related to sleep medicine or chronobiology during the basic cycle that the offered contents mainly covered elementary knowledge of sleep physiology (84.8%), neuroanatomical substrates of sleep and wakefulness (46.1%), and chronobiology of sleep and wakefulness (98.0%) (Figure 1A). During the clinical cycle, the students indicated that the offered contents mainly covered sleep hygiene (64.6%), insomnia (61.2%), and treatment of sleep disorders (57.9%), but there was less contact with sleep diagnostics and investigation (25.4%) (Figure 1B). During the internship, which was the current cycle of the respondents' program, they studied sleep medicine or chronobiology, particularly in disciplines such as psychiatry (28.7%), clinical medicine (20.7%), and family medicine (19.5%) (Figure 1C). Additionally, when asked about receiving guidance from professors on addressing sleep-related aspects during patient history taking (anamnesis) and diagnosis, 24 students (27.6%) reported not receiving such guidance, while 63 students (72.4%) reported having received guidance (Figure 1D).

Referring to all periods of their medical undergraduate program, when asked about the existence of any other mandatory core curriculum programs or elective modules dedicated to sleep medicine and chronobiology, only 9 students (3.7%) responded yes, with 6 being optional and 3 being mandatory (data not shown). In addition, when asked if sleep medicine or chronobiology content was covered in elective courses and at which period this occurred, 37 (15.4%) students responded yes, mainly during the basic cycle of the program (data not shown).

**Figure 2:**
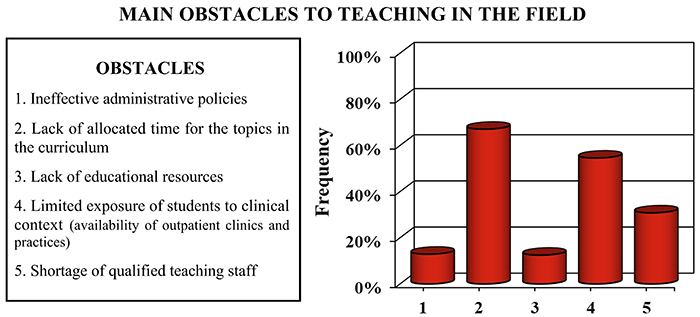
Obstacles for the effective teaching of chronobiology and sleep medicine as reported by medical students. The graph illustrates the percentage of the total number of students who reported each of the different obstacles they believe interfered with the effective teaching of chronobiology and sleep medicine. n=240 students.

When asked about barriers in the training in chronobiology and sleep medicine, 161 students (67.1%) cited insufficient dedicated time as the primary obstacle (Figure 2). Additionally, 131 students (54.6%) highlighted the insufficient immersion of students in clinical settings, including the low availability of outpatient clinics and practical experiences, as another significant challenge. Furthermore, minor issues such as a shortage of qualified faculty, inadequate educational resources, and ineffective administrative policies were also noted (Figure 2).

**Figure 3:**
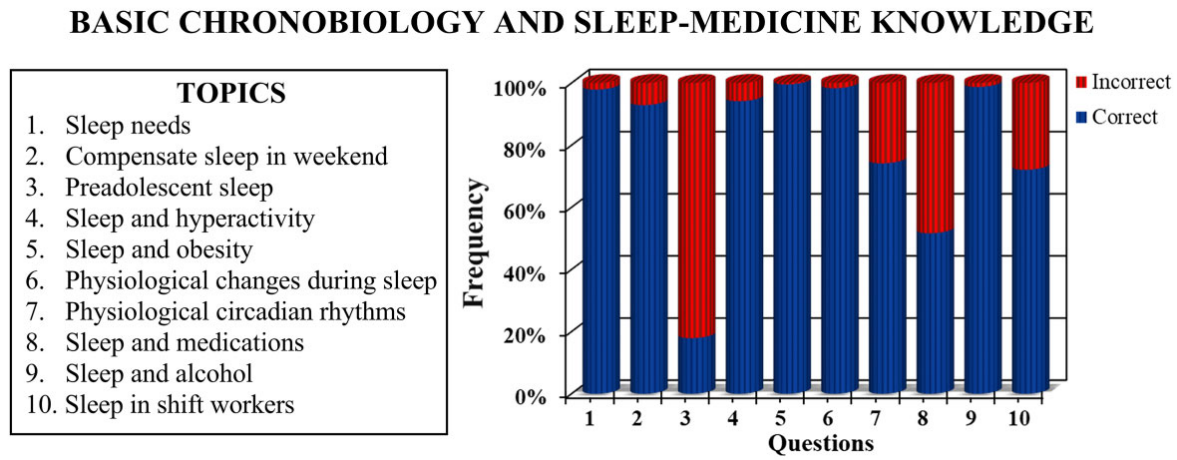
Frequency of correct and incorrect responses regarding different topics in chronobiology and sleep medicine. The panel describes the main topics associated with each of the 10 questions. n=240 students.

**Figure 4:**
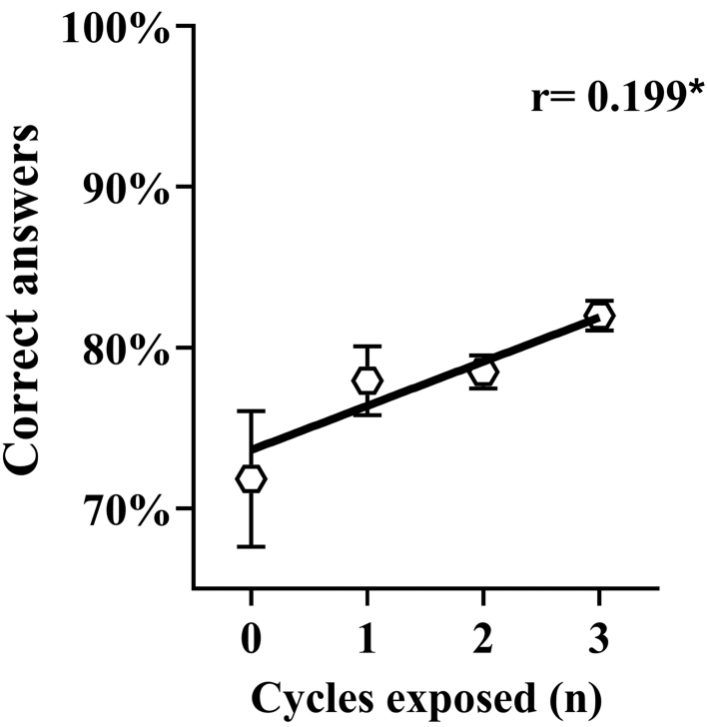
Spearman correlation analysis of the percentage of correct answers to the questions related to chronobiology and sleep medicine and the number of cycles with contact with the subject during the medical program. *P<0.05; n=240 students.

In the questionnaire of basic knowledge on sleep medicine and chronobiology, the average rate of correct answers was 79.75% (50-100%) across all questions. Notably, the highest error rates were observed in specific topics: sleep and pre-adolescence (with 82.08% of wrong answers) and the influence of drugs such as antihistamines and beta-blockers on sleep (with 48.33% of wrong answers) (Figure 3). Conversely, questions related to vital signs and circadian rhythms exhibited a moderate level of accuracy, with 25.83% of wrong answers, and those concerning work shifts and sleep had an error rate of 27.92%. Lastly, the remaining questions demonstrated a higher percentage of correct answers, ranging between 92.92 and 98.75% (Figure 3). Figure 4 shows a mild positive correlation between the number of cycles (including basic, clinical, and internship, or none) during which students were exposed to disciplines related to sleep medicine or chronobiology and the number of correct answers to corresponding questions.

The logistic regression analyses, both univariate and multivariate, indicated that a higher percentage of correct answers (≥80%) was not associated with most independent variables, such as sex, age, and country region (Table 3). However, a noteworthy association was found with academic year in the medical program, demonstrating that final-year students performed better than their counterparts in the fifth year (reference group). Additionally, despite the absence of an association with exposure to chronobiology or sleep medicine disciplines in each cycle of the medical program, the highest prevalence of correct answers was observed among students who engaged with these topics throughout all cycles (Table 3). Similar results were observed with multivariate analysis, indicating that the findings were not influenced by age, sex, country region, or academic year.

**Table 3:** Univariate and multivariate logistic regression analysis of performance of medical students in a questionnaire about chronobiology and sleep medicine.

Variable	N	Correct answers		Univariate OR (95%CI)	Multivariate^#^ OR (95%CI)
		No	Yes		
Age (years)					
18-24	125	79 (63.2%)	46 (36.8%)	Ref	Ref
>24	115	79 (68.7%)	36 (31.3%)	0.783 (0.458-1.338)	0.726 (0.413-1.278)
Gender					
Women	152	100 (65.8%)	52 (34.2%)	Ref	Ref
Men	88	58 (65.9%)	30 (34.1%)	0.995 (0.572-1.730)	1.031 (0.576-1.845)
Country region					
North	45	30 (66.7%)	15 (33.3%)	Ref	Ref
Northeast	87	53 (60.9%)	34 (39.1%)	1.283 (0.603-2.729)	1.235 (0.572-2.666)
Midwest	25	22 (88.0%)	3 (12.0%)	0.273 (0.070-1.059)	0.259 (0.066-1.022)
Southeast	48	32 (66.7%)	16 (33.3%)	1.000 (0.422-2.370)	0.856 (0.351-2.089)
South	35	21 (60.0%)	14 (40.0%)	1.333 (0.533-3.337)	1.406 (0.546-3.624)
Academic year					
5th	153	109 (71.2%)	44 (28.8%)	Ref	Ref
6th	87	49 (56.3%)	38 (43.7%)	1.921 (1.109-3.328)*	2.197 (1.224-3.942)*
Basic cycle^&^					
No	30	18 (60.0%)	12 (40.0%)	Ref	Ref
Yes	210	140 (66.7%)	70 (34.2%)	0.750 (0.342-1.644)	0.653 (0.285-1.495)
Clinical cycle^&^					
No	55	41 (74.5%)	14 (25.5%)	Ref	Ref
Yes	185	117 (63.2%)	68 (36.8%)	1.702 (0.865-3.347)	1.796 (0.888-3.633)
Internship^&^					
No	30	21 (70.0%)	09 (30.0%)	Ref	Ref
Yes	57	28 (49.1%)	29 (50.9%)	2.417 (0.946-6.173)	2.578 (0.939-7.075)
All cycles^&^					
No	33	24 (72.7%)	09 (27.3%)	Ref	Ref
Yes	54	25 (46.3%)	29 (53.7%)	2.016 (1.168-3.478)*	3.617 (1.292-10.126)*
Internship guidance					
No	24	15 (62.5%)	09 (37.5%)	Ref	Ref
Yes	63	34 (54.0%)	29 (46.0%)	1.422 (0.542-3.726)	0.962 (0.337-2.743)

^#^Multivariate regression analysis including age, gender, country region, and academic year as covariates; ^&^studies in chronobiology or sleep medicine during disciplines in the different cycles of medical program. *P<0.05 *vs* reference group. OR: odds ratio; CI: confidence interval.

## Discussion

In general, many medical students reported a low exposure to chronobiology and sleep medicine during their undergraduate studies. More specifically, studies in chronobiology and sleep medicine were more prevalent during the basic cycle of the medical program, characterized by an introductory approach to human physiology topics. However, the availability of these subjects decreased in subsequent years.

The investigative questionnaire revealed that studies in sleep medicine and chronobiology were offered to 210 students, constituting 87.5% of the total respondents, during the basic cycle, which corresponds to the initial two years of the medical undergraduate program and is the propaedeutic period for subsequent disciplines. During this period, sleep physiology and basic mechanisms were mentioned to a considerable extent, but the neuroanatomic substrates of sleep and wakefulness, as well as determinants of daytime sleepiness were covered only to a limited extent, indicating a deficiency in the teaching of sleep fundamentals. In the first two introductory years, important topics include sleep physiology, chronobiology of sleep and wakefulness, neuroautonomic substrates of sleep, dream studies, respiratory sleep parameters, sleep-related history and physical examination, followed by the pathophysiology of sleep disorders (19).

Consequently, during the clinical cycle, which extends over the two years following the basic cycle, students are engaged in clinical diagnostic and therapeutic reasoning through case studies. At this stage, only studies related to sleep investigation and diagnosis, as well as sleep disorders in children were mentioned. The data reveal a significant gap in the teaching of these subjects, which could lead to underdiagnosed patients and potentially erroneous treatment for non-sleep-related disorders in the future (20). In this context, the years of clinical training offer opportunities to integrate sleep-related topics, given that patients with sleep disorders present a range of symptoms, and these symptoms may reflect underlying primary disorders; for example, insomnia may manifest as a symptom of depression (21).

Internship is a mandatory cycle during which students undergo hospital and outpatient rotations in major medical areas such as internal medicine, surgery, gynecology and obstetrics, pediatrics, public health, and mental health. During this period, there is a greater emphasis on studies related to sleep medicine, particularly in the field of psychiatry. This might imply a bias toward a psychiatric diagnosis; however, it is important to acknowledge that sleep disorders can arise from metabolic, cardiovascular, neurological, immunological, or social factors (22). During these rotations, it is essential to provide students with guidance on diagnostic methods for sleep disorders, and consideration could be given to developing an elective module in sleep medicine to offer an intensive experience in the field (23).

Considering the population exposed to the topic during undergraduate studies, there was limited time dedicated to the study of sleep-related subjects (Figure 2). In line with this, a study involving 12 countries (Australia, India, Indonesia, Japan, Malaysia, New Zealand, Singapore, South Korea, Thailand, United States, Canada, and Vietnam) reported that the average amount of time spent on sleep education is slightly under 2.5 h, with 27% reporting no sleep education in their medical schools (24). Similarly, less than 2 h are allocated to teaching sleep and sleep disorders at 126 medical schools in the USA (10). Although sleep medicine teaching for undergraduate students in the UK has increased to an average of 1.5 h, a six-fold improvement compared to 1988, it is still considered insufficient (9).

In contrast, a recent study involving final-year medical students from seven Lebanese medical schools reported that higher scores on the ASKME were associated with sleep medicine education in the medical school curriculum (14). This is supported by the Institute of Medicine (IOM) report, recommending that exposure to sleep medicine should begin before entering residency and be integrated early into medical school curricula (25). In this way, understanding the rhythms related to the functions being assessed, especially the sleep-wake cycle, will assist the clinicians in their practice, enabling them to be more attentive to complaints involving their patients' rhythms. This expanded view provides the benefit of enlightening and guiding the patient about their life and work routines, especially regarding sleep disorders (26).

In addition to the benefits to the doctor-patient relationship, education in sleep medicine at the undergraduate level contributes to medical students' understanding of the health-disease process, particularly concerning chronobiological aspects and sleep health (27). Various studies have reported an association between poor sleep quality and both low academic performance and mental health in medical students (28-30). Additionally, a well-documented correlation exists between sleep disorders and suicidal behavior in both young people and adults (31).

The topic is likewise not a priority within other academic disciplines. A study conducted by our group involving 1,384 psychologists in Brazil, revealed a lack of familiarity with the term “chronobiology” and other biological rhythms beyond the sleep-wake cycle (15). Similarly, physiotherapists receive limited education on sleep (32), as do dentists, where education in dental sleep medicine in academic institutions in the USA and Canada faces numerous obstacles (33). In Brazil, concerning the general population, educational exposure to sleep-related subjects is scarce, especially considering the high prevalence of poor sleep quality associated with a lack of circadian and sleep hygiene practices (34).

The examination of data in this study provides insights into the distribution of content related to chronobiology and sleep medicine across undergraduate programs in Brazilian medical schools. The majority of respondents acquired knowledge in this field when exposed to the subject in three distinct cycles of their undergraduate medical education. Among this cohort, a significant variation in the number of correct answers was observed compared to students who encountered the subject at only one or two cycles or had no exposure at all.

Upon conducting a more detailed analysis of the correct answers, it is crucial to prioritize questions that displayed considerable variation, both positively and negatively. A thorough examination of responses indicated that question 3 showed a significant error rate (82.08%). This particular question is about the recognition that pre-adolescents and adolescents require more sleep and often have a vespertine chronotype, potentially leading to sleep deprivation due to early morning school schedules (35). The same question, when answered by medical students from Saudi Arabia and Lebanon, yielded error rates of approximately 50 and 65%, respectively (12,14). This disparity suggests that Brazilian students may have more limited knowledge of specific topics.

Although the continuity in exposure allows for the gradual consolidation of knowledge over time, enabling students to progressively deepen their understanding and skills, this finding contrasted with a significant number of schools that report no structured teaching time in this field, and only a minimal percentage of medical students receive training in sleep laboratory procedures or participate in the clinical evaluation of sleep-disordered patients (10). The data is in line with the notion that multiple exposures, as opposed to a single exposure, are more effective in facilitating a comprehensive understanding and application of knowledge, as demonstrated by the study of Marinopoulos et al. (36) on the effectiveness of continuing medical education.

The aforementioned observation also aligns with the conclusions from a comprehensive survey of USA medical schools, where significant impediments were reported. The identified obstacles encompassed the inadequacy of qualified faculty, limited curriculum time, and a demand for additional clinical and educational resources in the realm of sleep and sleep disorders education (10), as also declared by the students in the present study.

This study had several limitations. The low response rate may limit the generalizability of the findings, influenced by individual recruitment and social network invitations. To address the low response rate, we reduced the original ASKME (18) questionnaire from thirty to ten general questions, which may have introduced bias. Additionally, the lack of a validated Brazilian Portuguese version of the ASKME survey may impact its accuracy in reflecting the cultural and educational context of Brazilian medical students. The study also lacks curriculum evaluations and data on time allocated to the field, particularly given diverse approaches such as problem-based learning (PBL), which is organized into thematic modules across institutions. Furthermore, including only students from the final two years of medical school may have affected the analysis, as some students may still be in the early stages of their final years without complete exposure to chronobiology and sleep medicine, potentially influencing their knowledge level and response accuracy. Lastly, the absence of questions about self-directed study limits insights into the impact of independent learning on knowledge perception. These limitations suggest caution in interpreting the results and underscore the need for more comprehensive future research.

In conclusion, the study can serve as a cornerstone for future research aiming to expand the study of sleep medicine and chronobiology in medical schools by providing an overview of the Brazilian situation regarding the teaching of these topics. The strengths of the study include the use of a validated instrument (ASKME) for assessing sleep medicine knowledge and the inclusion of participants from both public and private medical education systems in the country, covering all 27 Brazilian states. The study provides insight into a previously unknown scenario regarding the teaching of sleep medicine in Brazilian medical schools.

## Supplementary Material

Click to view [pdf].
