# Supplementary material for: Sleep medicine and chronobiology education among Brazilian medical students
Source: Braz J Med Biol Res. 2024 Dec 13;57:e14147. doi: 10.1590/1414-431X2024e14147 (PMC11653483; doi:10.1590/1414-431X2024e14147)
Supplement: Supplementary file 1 [file 1414-431X-bjmbr-57-e14147-suppl.pdf]

Table S1. Questionnaire in English.

I. Student data

1) Age (years old): ☐ 18–24 ☐ >24

2) Sex: ☐ Men. ☐ Women.

3) Are you a student in the last two years of medical school? ☐ Yes ☐ No

4) Academic year: ________

5) The name of the institution where you study: ___________________

6) Brazilian state of the academic institution: __________

II. Research data

During which period of your college studies and in which subjects do you recall studying about Sleep Medicine and Chronobiology?

1) Basic cycle: ( ) 1st year ( ) 2nd year

a. In which discipline or area of knowledge did you encounter the topic?

☐ Neural science ☐ Physiology ☐ Behavioral Science ☐ Psychiatry
☐ Family medicine ☐ Other. Which? _________.

2) Clinical cycle: ( ) 3rd year ( ) 4th year

a. In which discipline or area of knowledge did you encounter the topic?

☐ Medical clinic ☐ Neurology ☐ Psychiatry ☐ Pediatrics
☐ Family medicine ☐ Other. Which? _________.

3) Elective course: ( ) 1st year ( ) 2nd year ( ) 3rd year ( ) 4th year

4) Internship: ( ) 5th year ( ) 6th year

a. In which discipline or area of knowledge did you encounter the topic?

☐ Medical clinic ☐ Neurology ☐ Psychiatry ☐ Pediatrics
☐ Family medicine ☐ Other. Which? _________.

5) Were you instructed to address sleep in taking the patient's medical history (anamnesis) and diagnosis? ______________

6) Do you recall any other programs or modules dedicated to sleep (optional): number / period it occurred / mandatory or optional? ___________________.

7) What subtopics do you remember having covered in the basic cycle?

☐ Physiology of sleep and basic mechanisms ☐ Neuroanatomical substrates
☐ Sleep/wakefulness chronobiology ☐ Pharmacology of sleep/wakefulness
☐ Determinants of daytime sleepiness ☐ Developmental aspects of sleep
☐ Phylogenetic aspects of sleep ☐ Sleeping and dreaming
☐ Others: ______________

8) What subtopics do you remember having covered in the clinical cycle?

☐ Respiratory conditions during sleep ☐ Sleep disorders in psychiatry

- Treatment of sleep disorders
- Sleep disorders in clinical practice
- Hypersomnia (non-respiratory)
- Parasomnia
- Sleep hygiene
- Insomnia
- Circadian rhythm disorder
- Sleep diagnostics and investigation
- Sleep disorders in children
- Others: _______________

9) Do you believe there are obstacles to effective teaching of these subjects? If yes, what are they?

- Lack of time allocated to the subjects in the curriculum
- Lack of qualified teaching staff
- Lack of educational resources
- Lack of student immersion in clinical context (lack of outpatient clinics and practical experiences)
- Ineffective administrative policies

III. Questionnaire on Basic Knowledge in Sleep Medicine and Chronobiology

1) The need of sleeping hours varies according to age.

☐ True ☐ False ☐ Don't know

2) It is recommended to compensate for sleep loss during the week by sleeping more on weekends.

☐ True ☐ False ☐ Don't know

3) Pre-adolescents with consistent sleep-related problems should be allowed to sleep more in the morning.

☐ True ☐ False ☐ Don't know

4) Childhood hyperactivity can worsen with inadequate sleep.

☐ True ☐ False ☐ Don't know

5) Obesity can be a factor for primary snoring or obstructive sleep apnea.

☐ True ☐ False ☐ Don't know

6) Heart rate, respiratory rate, and blood pressure vary in relation to the sleep period compared to wakefulness.

☐ True ☐ False ☐ Don't know

7) Heart rate, respiratory rate, and blood pressure exhibit circadian rhythms.

☐ True ☐ False ☐ Don't know

8) Antihistamines can induce sleep, while beta-blockers can cause difficulty sleeping.

☐ True ☐ False ☐ Don't know

9) Alcohol consumption interferes with sleep and potentially its quality.

☐ True ☐ False ☐ Don't know

10) Night shift workers are more likely to fall asleep at work compared to workers with regular daytime shifts.

□ True □ False □ Don't know

Table S2. Questionário em Português brasileiro.

I. Dados do estudante

1) Idade (anos): ☐ 18–24 ☐ >24

2) Sexo: ☐ Masculino. ☐ Feminino.

3) É aluno dos dois últimos anos de graduação em medicina? ☐ Sim ☐ Não

4) Ano acadêmico: ________

5) Nome da Instituição: ____________________

6) Estado em que estuda: __________

II. Dados da pesquisa

Em qual período da faculdade e matérias você se recorda ter estudado sobre Medicina do Sono e Cronobiologia?

1) Ciclo básico: ( ) 1º ano ( ) 2º ano

a. Em qual disciplina ou área do conhecimento você viu o assunto?

☐ Ciência neural ☐ Fisiologia ☐ Ciência comportamental ☐ Psiquiatria

☐ Medicina de Família ☐ Outro. Qual? _________.

2) Ciclo clínico: ( ) 3º ano ( ) 4º ano

a. Em qual disciplina ou área do conhecimento você viu o assunto?

☐ Clínica Médica ☐ Neurologia ☐ Psiquiatria ☐ Pediatria ☐ Medicina de Família

☐ Outro. Qual? _________.

3) Eletiva: ( ) 1º ano ( ) 2º ano ( ) 3º ano ( ) 4º ano

4) Internato: ( ) 5º ano ( ) 6º ano

a. Em qual disciplina ou área do conhecimento você viu o assunto?

☐ Clínica Médica ☐ Neurologia ☐ Psiquiatria ☐ Pediatria ☐ Medicina de Família

☐ Outro. Qual? _________.

5) Você foi orientado a abordar o sono na construção da anamnese (história clínica) e do diagnóstico do paciente? ______________

6) Se recorda de algum outro programa ou módulos dedicados ao sono (opcional): número / período em que acontece / obrigatório ou facultativo? ___________________.

7) De quais subtópicos você lembra de ter tido no ciclo básico?

☐ Fisiologia do sono e mecanismos básicos ☐ Substratos Neuroanatômicos

☐ Cronobiologia do sono / vigília ☐ Farmacologia do sono / vigília

☐ Determinantes da sonolência diurna ☐ Aspectos de desenvolvimento do sono

☐ Aspectos filogenéticos do sono ☐ Dormir e sonhar

☐ Outros:_______________

8) De quais subtópicos você lembra de ter tido no ciclo clínico?

☐ Condições respiratórias no sono ☐ Distúrbios do sono na psiquiatria

 Tratamento de distúrbios do sono
 Distúrbios do sono na clínica
 Hipersonias (não-respiratória)
 Parassonias
 Higiene do sono
 Insônia
 Desordem do ritmo circadiano
 Diagnósticos e investigação do sono
 Distúrbios do sono em crianças
 Outros: _________________

9) Acredita que existem obstáculos para o ensino efetivo dos assuntos? Se sim, quais?
 Falta de tempo destinado aos temas, no currículo
 Falta de corpo docente qualificado
 Falta de recursos educacionais
 Falta de imersão do aluno em contexto clínico (oferta de ambulatórios e práticas)
 Política administrativa inoperante

III. Questionário de conhecimento em Medicina do Sono e Cronobiologia

1) A necessidade de sono varia de acordo com a idade.
 Verdadeiro Falso Não Sei

2) Recomenda-se compensar a perda de sono durante a semana, dormindo mais durante os finais de semana.
 Verdadeiro Falso Não Sei

3) Pré-adolescentes com problemas constantes relacionados ao sono, deveriam poder dormir até mais tarde pela manhã.
 Verdadeiro Falso Não Sei

4) A hiperatividade infantil pode se agravar ainda mais com um sono inadequado.
 Verdadeiro Falso Não Sei

5) A obesidade pode ser fator para ronco primário ou para apneia obstrutiva do sono.
 Verdadeiro Falso Não Sei

6) A frequência cardíaca, frequência respiratória, e pressão arterial variam em relação ao período de sono comparado ao de vigília.
 Verdadeiro Falso Não Sei

7) A frequência cardíaca, frequência respiratória, e pressão arterial apresentam ritmos circadianos.
 Verdadeiro Falso Não Sei

8) Anti-histamínicos podem induzir sono, enquanto betabloqueadores podem causar dificuldade para dormir.
 Verdadeiro Falso Não Sei

9) A ingestão de bebida alcoólica interfere no sono e potencialmente na qualidade dele.

□ Verdadeiro □ Falso □ Não Sei

10) Trabalhadores de turno noturno adormecem mais facilmente no trabalho em comparação aos trabalhadores com turno regular e diurno.

□ Verdadeiro □ Falso □ Não Sei
